# Trends in Peruvian scientific publications on COVID-19: A bibliometric analysis

**DOI:** 10.1590/1516-3180.2020.035322072020

**Published:** 2020-08-14

**Authors:** Vania Alexandra Tellez, Walter Andree Tellez

**Affiliations:** I Medical Student, Universidad Ricardo Palma, Lima, Peru.; II MD. Physician, Universidad Nacional Federico Villarreal, Lima, Peru.

Dear Editor,

The coronavirus disease 2019 (COVID-19), which was declared to be a pandemic by the World Health Organization (WHO) on March 11, 2020, is an important issue worldwide.^1^ Because of the importance of increasing knowledge about COVID-19, some initiatives have been set up to encourage scientific research in this field in Peru. The National Council for Science, Technology and Technological Innovation (CONCYTEC) generated calls for funding on COVID-19 research. Similarly, the Peruvian Journal of Experimental Medicine and Public Health (Revista Peruana de Medicina Experimental y Salud Pública, RPMESP) called for manuscripts on COVID-19 to be included in its next issues. However, no bibliometric study on Peruvian scientific production relating to COVID-19 has yet been conducted. Therefore, we resolved to perform a bibliometric analysis in this field.

We conducted a systematic search for Peruvian scientific articles on COVID-19 in the PubMed/MEDLINE and SciELO databases, and directly in the RPMESP archives up to May 21, 2020. The search strategy for PubMed/MEDLINE was (COVID-19 [MeSH] OR COVID-19 diagnostic testing [MeSH] OR COVID-19 drug treatment [MeSH] OR COVID-19 serotherapy [MeSH] OR COVID-19 vaccine [MeSH] OR severe acute respiratory syndrome coronavirus 2 [MeSH] OR COVID-19 [Title/Abstract] OR SARS-CoV-2 [Title/Abstract] OR coronavirus disease 2019 [Title/Abstract] OR Wuhan coronavirus [Title/Abstract]) AND (Peru [MeSH] OR Peru [Title/Abstract] OR Peruvian [Title/Abstract] OR Andean [Title/Abstract]). For SciELO, it was (COVID-19) OR (infections coronavirus) OR (coronavirus disease 2019) OR (SARS-CoV-2). Articles that did not provide scientific knowledge were excluded.

We included 24 articles (Figure 1). Eleven articles (45.83%) were published in RPMESP. Only 12.50% were published in Q1 journals; 29.17% were original and/or brief reports; and 66.67% were indexed in PubMed/MEDLINE and Scopus (Table 1). Most of the authors (91.70%) had affiliations with institutions in Lima, Peru. The highest numbers of publications were attributed to Universidad Nacional Mayor de San Marcos (UNMSM) (25.00%) and to Universidad Peruana Cayetano Heredia (UPCH) (20.83%).

**Figure 1.**
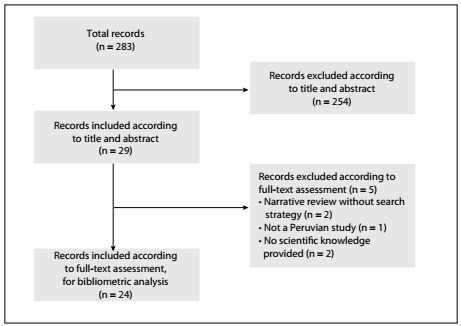
Flowchart of study selection.

**Table 1.** Bibliometric characteristics of Peruvian scientific articles on COVID-19

	n (%)
Journal name	
Psychiatry Research	1 (4.17)
Travel Medicine and Infectious Disease	1 (4.17)
AIDS and Behavior	1 (4.17)
Revista de la Facultad de Ciencias Médicas de Córdoba	1 (4.17)
Revista de Gastroenterologia del Perú	1 (4.17)
Microbiology Resource Announcements	1 (4.17)
Revista Peruana de Medicina Experimental y Salud Pública	11 (45.83)
Pre-print	6 (25.00)
Quartile	
Q1	3 (12.50)
Q2	0 (0.00)
Q3	11 (45.83)
Q4	2 (8.33)
NA	8 (33.33)
Language	
Spanish	19 (79.17)
English	5 (20.83)
Foreign collaboration	
Yes	5 (20.83)
No	19 (79.17)
Number of participating institutions per article (M ± SD)	2.75 ± 1.54
Type of article	
Original	6 (25.00)
Brief report	1 (4.17)
Case report	1 (4.17)
Letter to editor/correspondence	7 (29.17)
Review article	3 (12.50)
Editorial	5 (20.83)
Other	1 (4.17)
Indexing databases	
PubMed/MEDLINE	
Yes	16 (66.67)
No	8 (33.33)
Scopus	
Yes	16 (66.67)
No	8 (33.33)
SciELO	
Yes	18 (75.00)
No	6 (25.00)

NA = not available; M = mean; SD = standard deviation.

The topics addressed mostly were related to epidemiology, with emphasis on the probability of effectiveness or failure regarding pandemic control measures. The technological contribution of telemedicine and tele-education was also addressed. In addition, research on mental health in the era of COVID-19 pointed out the possibility of undesired outcomes such as development of psychotic symptoms due to inadequate management of anxiety caused by COVID-19.

We found that around 10% of the articles were published in Q1 journals. Scientific publication in higher-quartile journals increases the likelihood of citation because of higher impact factors and h-indexes, which are important indicators of scientific-academic publication.^2^ Therefore, Peruvian researchers should aim to publish more frequently in those journals. Moreover, about 30% of the articles were original. This is the main type of publication in scientific journals, and forms an important indexing criterion in different databases.

In Peru, UNMSM and UPCH provide the highest scientific production within medicine.^3^ Accordingly, the largest proportion of COVID-19 publications was also attributed to those universities.

In conclusion, Peruvian scientific production on COVID-19 has emphasized epidemiological research, and has mainly been produced within institutions located in Lima. Therefore, there is a need to decentralize collaborative networks and promote clinical studies in order to generate more evidence with which to combat the pandemic.

## References

[1] Hamid S, Mir MY, Rohela GK (2020). Novel Coronavirus Disease (COVID-19): A Pandemic (Epidemiology, Pathogenesis and Potential Therapeutics). New Microbes New Infect.

[2] Grech V, Rizk DEE (2018). Increasing importance of research metrics: Journal impact factor and H-index. Int Urogynecol J.

[3] Mayta-Tristán P, Toro-Huamanchumo CJ, Alhuay-Quispe J, Pacheco-Mendoza J (2019). Producción científica y licenciamiento de escuelas de medicina en el Perú. Rev Peru Med Exp Salud Publica.

